# Basophilic Stippling and Chronic Lead Poisoning

**DOI:** 10.4274/tjh.2018.0195

**Published:** 2018-11-13

**Authors:** Yantian Zhao, Juan Lv

**Affiliations:** 1 Capital Medical University Beijing Chao-yang Hospital, Department of Clinical Laboratory, Beijing, China

**Keywords:** Basophilic stippling, Chronic lead poisoning, Blood

A 66-year-old female patient presented with a 9-month history of abdominal colic and fatigue that prompted an abdominal computed tomography scan and gastrointestinal endoscopy with negative assessments. The hemoglobin level was 72-81 g/L with an increase in serum ferroprotein. Bone marrow (Figure 1A) and peripheral blood smears (Figure 1B) revealed extensive erythrocytes with coarse basophilic stippling. It was suggested that there may have been an accumulation of heavy metal in her body. The level of lead in her blood and urine was increased to 1036 µg/L (permissible: <400 µg/L [1]) and 246 µg/L (permissible: <70 µg/L [1]), respectively. Her blood mercury level was below the permissible level (permissible: <15 µg/L [2]). Therefore, she was initially diagnosed with chronic lead poisoning. Further history revealed that she had been taking an adulterated dietary supplement called “Fengwangjiang” from unverified sources for more than the past 1 year. Bone marrow smears showed 4+ iron stores (Figure 1C) and ring sideroblasts (Figure 1D), indicating ineffective heme synthesis. She complied with the doctor’s order to stop taking the dietary supplement and received lead-chelation therapy during hospitalization, and her symptoms improved. Basophilic stippling provides a clue to the underlying diagnosis and an understanding of the underlying pathogenesis.

## Figures and Tables

**Figure 1:**
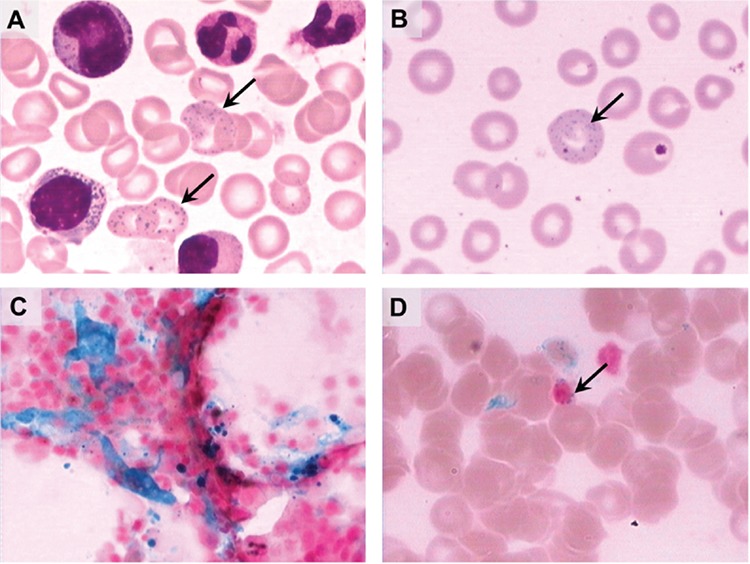
(A) Bone marrow smears and (B) peripheral blood smears revealing extensive erythrocytes of coarse basophilic stippling (1000^x^, Wright-Giemsa stain). Bone marrow smears showed 4+ iron stores (C) and ring sideroblasts (D) (1000^x^ iron stain).
